# Beyond dichotomies: Gender and intersecting inequalities in climate change studies

**DOI:** 10.1007/s13280-016-0825-2

**Published:** 2016-11-22

**Authors:** Houria Djoudi, Bruno Locatelli, Chloe Vaast, Kiran Asher, Maria Brockhaus, Bimbika Basnett Sijapati

**Affiliations:** 1 CIFOR, Situ Gede, Sindang Barang, Bogor Bar, Jawa Barat Indonesia; 2 CIRAD-CIFOR, Avenida La Molina 1895, Lima 12, Peru; 3 The Royal Tropical Institute (KIT), Mauritskade 63, 1092 AD Amsterdam, The Netherlands; 4 University of Massachusetts, 208 Bartlett Hall, Amherst, MA 01003-9717 USA; 5 Jalan CIFOR, Situ Gede, Sindang Barang, Bogor Bar, Jawa Barat Indonesia

**Keywords:** Adaptation, Climate change, Gender, Intersectionality

## Abstract

Climate change and related adaptation strategies have gender-differentiated impacts. This paper reviews how gender is framed in 41 papers on climate change adaptation through an intersectionality lens. The main findings show that while intersectional analysis has demonstrated many advantages for a comprehensive study of gender, it has not yet entered the field of climate change and gender. In climate change studies, gender is mostly handled in a men-versus-women dichotomy and little or no attention has been paid to power and social and political relations. These gaps which are echoed in other domains of development and gender research depict a ‘feminization of vulnerability’ and reinforce a ‘victimization’ discourse within climate change studies. We argue that a critical intersectional assessment would contribute to unveil agency and emancipatory pathways in the adaptation process by providing a better understanding of how the differential impacts of climate change shape, and are shaped by, the complex power dynamics of existing social and political relations.

## Introduction

Climate change will affect people differently according to their cultural, economic, environmental and social context. A number of studies raise the need to recognize the importance of social differentiation as a crucial determinant of vulnerability (Adger and Kelly 1999; O’Brien et al. 2007; Ribot 2010, Tschakert 2012). The integration of a social science perspective in climate change occurs slowly and the feminist research perspective on climate change occurs even more slowly. Without a “sociology of climate change” (MacGregor 2010, p. 137), we will not be able to understand the root causes of the climate crisis and will fail to tackle global warming. People and groups are situated within broader socio-cultural, political and economic relations. Indeed, the capacity to adapt and respond to change is shaped by power relations determining access to resources, information and the availability of options and choices (Tschakert 2012; Djoudi et al. 2013). These factors are related to the social identities and positions of people and groups. Gender is a key element of these identities and relations. Furthermore, vulnerability and adaptive capacity are dynamic in nature, and changes affecting them at one level can have profound and hidden implications at other levels (Pelling 2010). Thus, if the root causes of vulnerability are not taken into account, potential solutions might exacerbate rather than reduce existing injustices, while leaving challenges of climate change unaddressed (MacGregor 2010). Concerns about women and gender within the climate change literature need to be located within this context.

In the fields of vulnerability analysis, it is crucial, in Lykke’s (2009, p. 43) words, “to bring more forcefully the issues of complicity, tensions and conflicting interests not only between ‘the vulnerable’ and hegemonic powers, but also among ‘the vulnerable’ themselves”. Recent climate change studies highlight that: “Women are once again being singled out as climate victims” or are usually framed as “victims or stewards” (Arora-Jonsson 2011). Furthermore, simplistic dichotomies fail to capture the range of complexities and the power dynamics of vulnerability. This is often because “women’s identities are projected as fixed, centred, and uniform” (Resurrección 2013, p. 1), ignoring that other factors like age, wealth, class and ethnic affiliation are often crucial (Djoudi and Bockhaus 2011). Earlier feminist work, for instance Tuana (1993) called for more scepticism towards dichotomies and for more gender approaches which overcome dualism.

This paper draws on the insights of this recent scholarship, and the analytical frameworks of intersectionality and vulnerability, to argue for a more nuanced understanding and analysis of gender in the climate change debate. We first outline the frameworks and approaches that inform our analyses. Next we discuss how gender is currently addressed in the scientific literature on climate change. This is followed by a discussion of our findings and our conclusions.

### Theorizing the linkage between gender, climate change and adaptation

This study aligns itself with theories on gender and climate change to analyse the gender trends in the existing climate change literature. It relies on intersectionality as a tool for analysing climate change publications. Furthermore, this paper aims to bring together existing concepts (i.e. intersectionality, vulnerability) to critically assess and enrich common climate change and gender concepts and theories.

### Intersectionality

The concept of intersectionality can address some of the important issues in the debates on vulnerability and adaptive capacity to climate change. The term intersectionality was first used in the early 1990s in the field of critical race theory to respond to binary gender analysis (women/men). Based on Kim Crenshaw’s (1991) work on intersecting legal identities, intersectionality provides a more complex ontology than approaches that attempt to reduce people to a single category at a time. Intersectionality is based on the assumption that social categories (i.e. ‘race’/ethnicity, gender, class, sexuality and ability) are constructed and dynamic (Davis 2008; Cho et al. 2013; Kaijser and Kronsell 2014). Furthermore, “Intersectionality conceptualizes social categories as interacting with and co-constituting one another to create unique social locations that vary according to time and place” (Hankivsky 2012, p. 35).

Drawing on a long history of feminist approaches, in intersectional analysis, the relational nature of power is important, since the same subject can experience both power and oppression simultaneously (Collins 1990). Intersectionality calls for a nuanced analysis of power “that includes not only power over others, but also power with others” (Hankivsky 2012, p. 36). Furthermore, this author argues that intersectionality calls on us to move beyond the ‘Oppression Olympics‘, a term used by Martinez (1993) to describe the “competition between different groups for the title of ‘most oppressed’, in order to gain support, economic resources, and recognition” (Hankivsky 2012, p. 36). Through the examination of the intersecting factors and conditions by which power is not only produced, reproduced but also actively resisted, intersectionality calls for a more complex approach to address the system that creates power differentials, rather than the symptoms of it (Hancock 2007). Hence, from an intersectional point of view, power structures in a specific social categorization system determine how individuals relate and react to climate change (Kaijser and Kronsell 2014). Furthermore, intersectionality enables people and communities to express and experience their own capacity because it creates a pathway of analysis enabling “agency across and beyond social categories” (Kaijser and Kronsell 2014, p. 417).

The multilevel dimension of intersectionality recognizes complex, horizontal (inter-community) and vertical (national, regional, local) interactions. It includes not only the impact of different policies at various levels, but also the inter-community asymmetric power relations based on social identities, or as a reaction to policies. Apart from the high potential of intersectionality to address some of the gender issues that are at stake in the climate change and other debates, we need to keep in mind that this concept is still ambiguous and there is a need to identify how the concept should be defined and to which levels should be appropriately applied (Davis 2008; Winker and Degele 2011). While intersectionality has not yet been applied in specific local studies in the field of climate change, we argue that it has the potential to address some misleading issues relating to climate change debate and analysis. By reflecting multifaceted power relations at different scales, intersectionality takes a step towards reflecting an equitable adaptation process. Besides identifying and analysing power, an intersectional analysis of climate change can help to unveil explicit and implicit assumptions about social categories and their relations (Kaijser and Kronsell 2014).

### Emancipation, agency and adaptation

While recognizing that the vulnerability and adaptation framework is helpful in portraying differential impacts of climate change, several authors argue that the concept may generate a restricted idea of vulnerability as a passive, innocent victimhood (Cannon 2008; Weisser et al. 2014). In this context, we argue that highlighting the importance of agency and emancipatory transformational pathways in the adaptation process can address some of those issues that are foremost in the debate on vulnerability and adaptation. We refer to emancipation in terms of Bourdieu’s concept of power and social space (Bourdieu 1989; Bourdieu and Thompson 1991). Power is not considered as being external to those on whom it is exercised. Instead, it is framed as a social relationship. This theoretical framework is crucial when establishing the necessary conditions for the process of emancipation.

In his essays on the ‘Spatialities of Emancipation’ and the ‘City of Thresholds’, Stavrides (2010, p. 39) points out that “The experience of temporarily occupying an in-between territory, as well as an in-between non-identity, can provide us with a glimpse of a spatiality of emancipation. Creating in-between spaces might mean creating spaces of encounter between identities instead of spaces characteristic of specific identities”. By its nature, the adaptation process could create such spaces, by the potential of shaking and challenging the social and cultural determinants of a society or a community. ‘Transit identities’ as described by Stavrides (2010), which might be engendered by those changes, are an important emancipatory space, apart from the fact that they do not necessarily result from negotiations between equals. The question here, to conclude this discussion of Stavrides’ work, is: can the threshold created by a crisis become the spatial equivalent of an emancipating pathway, based on negotiation between different identities in the process of collectively inventing the future?

There is extensive research on the various ways in which women are subject to, and actively resist, capitalist, patriarchal and other forms of subjugation (Kandiyoti 1988, 2005; Asher 2007). Within the agricultural production system of rural societies, social hierarchies based on combinations of lineage, gender and age determine resource access, division of labour and decision-making mechanisms. Women and youth are crucial to agricultural work, but their involvement in decision making and their access to assets are limited (Kabeer 1994; Fortmann and Rocheleau 1997; Colfer 2005; Gautier and van Santen 2014; White 2012).

Within the structure of colonial and postcolonial economies, control over resources and the claims of chiefs on the labour of women and youth (Chauveau and Richards 2008; White 2012) are still well reflected in “the institutional fabric of rural cash-cropping societies” (Chauveau and Richards 2008, p. 517).

While resistance, social movements and civil society pressures are known to influence the broader political economy that shapes household entitlements (Ribot 2010), little is known about how individuals, specifically women, shape those outcomes in the context of traditional hierarchical production systems in response to external major changes (e.g. climate, migrations). There is a need to explore adaptation in a broader social context, including its impacts on social structures. We argue that the recognition of unbalanced power relations must also necessarily involve acknowledging the resistance, contestation and emancipation patterns that are intertwined with power. However, few studies document the complex reactions and mechanisms that challenge and push social barriers in an attempt to create space for emancipatory pathways. To understand adaptation at the local level, we need to study the nature of reactions, their possible collective or individual emancipatory elements and the social objectives and changes they might be conceptualized for. Access and rights to natural resources represent some of the many ways that inequities materialize. We therefore argue that emancipation strategies can be reflected in changes in patterns of natural resource use throughout the adaptation process.

In this study, we apply an intersectionality lens to analyse the existing climate change studies, and identify potential gaps in the recognition of emancipation and agency as an important part of adaptation strategies, and to discuss their implications.

## Approaches and methods

### Literature search and the applied intersectionality framework

We searched for literature on climate change, rural livelihoods and gender on 12 March 2013 using several databases (Web of Knowledge, SCOPUS, Wiley, Science Direct, Agricola, Agris, CAB Abstracts, Econlit, Pascal, and Francis). We used queries composed of five groups of keywords related to climate, rural livelihoods, mitigation, adaptation and gender (Table 1). The OR operator connected keywords within groups and the AND operator connected keyword groups.

**Table 1:** Keywords used in the literature search

Group	Terms
Study scope (climate change and rural livelihoods)	Climate* AND (society OR “rural communit*” OR “local communit*” OR “local people” OR livelihood* OR “Indigenous people” OR “community household*” OR “rural household*” OR “farmer household*” OR “forest household*” OR “farming communit*” OR farmer* OR fisherfolk OR fisherm* OR “livestock keeper*” OR rancher* OR “forest dweller*” OR “forest people*”)
Mitigation	Mitigation OR REDD OR “Emissions from Deforestation” OR CDM OR “Clean Development” OR “carbon project*” OR “carbon payment*” OR “carbon offset*” OR “carbon sequestration” OR “carbon storage” OR “carbon absorption”
Adaptation	Adapt* OR vulnerab* OR resilien* OR coping OR cope
Gender	Gender OR women OR “female villager*” OR “female headed” OR “female farmer*” OR “female member*”

After removing duplicates, the search of all databases resulted in 345 papers. After screening titles and abstracts, we selected 196 potentially relevant papers. After reading through the papers and removing irrelevant papers (e.g. papers mentioning gender, climate change adaptation and mitigation only in passing, or papers that are conceptual but do not include case studies), we selected a final list of 41 papers on adaptation and 15 on mitigation. The adaptation papers dealt with gender in the context of climate variability in the short term (e.g. droughts) and climate change in the long term (e.g. gradual changes in rainfall). Given the small number of selected papers on mitigation, we decided to focus on papers on adaptation.

#### The intersectionality framework

There is a growing body of literature discussing the theoretical aspects of intersectionality. Hankivsky (2012) developed and applied an intersectionality-based policy analysis framework for the health sector. However, very few studies focus on the application of intersectionality to the specific field of climate change. Kaijser and Kronsell (2014) developed specific question to sensitize researchers to the integration of the intersectional analysis on climate change. Based on the insights those studies provide, we aimed to contribute to developing a proper framework for intersectional analysis of climate change. This framework has been tested in the analysis of the climate change studies and will be assessed and finalized in another publication. For this study, we identified and focused on three specific aspects of the intersectionality framework that are relevant for vulnerability (Table 2). In the context of unbalanced power relations, it is common wisdom that the impact of climate change may reinforce existing inequalities. We argue however that this assumption is associated with the concepts of victimhood and passiveness of the ‘vulnerable’. We aim to analyse these selected papers in light of unexpected social outcomes that challenge gender, ethnicity, caste, etc.; other existing structures might also emerge in this process. Hence, to explore this field, we added a fourth dimension to the framework that focuses on an analysis of emancipation pathways and agency so as to include emancipation patterns as an important but often disregarded part of adaptation strategies.

**Table 2:** Intersectionality dimensions considered and guiding questions for the review

Dimension of intersectionality	Guiding questions applied to code the articles
Intersecting categories	1. Which social categories where included in the vulnerability analysis? 2. Was the intersection of different social and cultural factors in vulnerability dynamics and power relations considered?
Multilevel analysis	3. Did the analysis include vulnerability impacts and dynamics affecting institutions and relationships across various levels of society? 4. Did the vulnerability analysis include intra-community and intra-household inequities?
Power	5. How was power framed in the papers? 6. Did the paper analyse how power and inequity are produced, reproduced and actively resisted (Hankivsky 2012)? 7. Did the paper analyse how climate change adaptation processes shape power relations with reference to social determinants (gender, class, race, etc.)?
Emancipation patterns, agency and resistance	8. Are they results from case studies that document changes in power relations and social structures associated with environmental change and the social process of adaptation to it? 9. Whether, and to what extent, do adaptation processes reinforce or challenge hierarchical, unequal social structures? 10. Did the paper refer only to vulnerability or did it include references to agency across and beyond existing social categories? 11. Did the paper identify any changes challenging the dynamics of gender, ethnicity, caste, age, etc.? 12. Did the results of the paper include evidence of resistance towards social norms?

#### The vulnerability and adaptive capacity framework

To understand how the gender aspects of climate change are approached, we broadened and built on the vulnerability framework (Table 3).

**Table 3:** Guiding questions for the review of vulnerability and adaptive capacity

Guiding questions used for the data analysis and to code the articles	
1. Did the paper include in its gender rationale how different gender groups are exposed differently to climate variations and change? 2. Did the paper differentiate impacts and/or perceptions of climate change according to gender? 3. How many papers have looked specifically at vulnerabilities of marginalized groups? 4. How many papers analysed gendered vulnerabilities based on case study data/evidence? 5. What are the factors included in the analysis to understand the differential vulnerability of different social groups? Did the paper include an analysis of the capabilities of different groups to cope with extreme events? 6. Did the paper identify specific adaptive strategies established by different social groups? 7. Did the paper provide specific recommendations for different social groups?	

## Results

### The nature of the data and the relevance of scale

Our results show that, while the scientific literature attempts to integrate gender in the study of climate change adaptation and mitigation, there are still gender-biased assumptions and knowledge gaps. For instance, gender was addressed less frequently in studies on mitigation than in those on adaptation. The lack of articles addressing gender issues and climate mitigation may be due to the prevailing notion in the mitigation debate that scientific and technological solutions are generally considered to be a male domain (MacGregor 2010), often at the expense of social and behavioural considerations (Aguilar 2010). Table 4 summarizes the results in terms of the frequency of the gender-relevant approaches as found in the analysed publications.

**Table 4:** Frequency of gender-relevant approaches and findings in the reviewed papers

Gender-relevant aspects of the reviewed papers	Frequency^a^
Approach or framework	
Paper based on case studies	***
Findings	
Intersectionality	
Consideration of two categories (i.e. men and women)	****
Consideration of age as a variable in addition to gender in the analysis	***
Consideration of ethnicity as a variable in addition to gender in the analysis	***
Consideration of profession as a variable in addition to gender in the analysis	***
Consideration of wealth as a variable in addition to gender in the analysis	**
Focus on differentiated perceptions of exposure and impacts, rather than differentiated vulnerability	–
Use of equity and rights-based perspectives as a rationale for gender integration	*
Analysis of social and political power relations	–
Consideration of existing intersectional inequalities	–
Agency and emancipatory pathways	
Adaptation to climate change leads to social shift in relation to gender	***
Women are adaptable and play an important role in household adaptation	***
Men and women have different coping or adaptation strategies	***
Adaptive strategies have gender-differentiated outcomes	***
Migration is one of a number of male-dominated strategies expected to impact gender relationships	**
Consideration of women’s agency, active choices and engagement	*
Men and women perceive different adaptation needs	*
Men and women play different roles in the implementation of one specific adaptation activity	*
Vulnerability and adaptive capacity	
Divergent perceptions are explained by gendered livelihood activities, roles and responsibilities	****
Assets and context increase vulnerability and barriers to adaptation for women	****
Assumption or general statement that women are more vulnerable than men	***
Focus on the perceptions of climate variations, rather than their implications for the vulnerability of individuals or households	**
Men and women have different perceptions of climate variations, their causes and impacts	**
Women and men are impacted differently by climate variability	**
Evidence that women are more vulnerable than men based on case studies at the local level	*
Vulnerability of female-headed households is evidenced	*
Consideration of differentiated intra-households vulnerabilities	*
Divergent perceptions are explained by women’s vulnerability	*

^a^Refers to the frequency of this approach or to findings in the reviewed papers: no papers: “–”, * Very few papers: less than 10 % of papers, ** few papers: from 10 % to less than 20 %, *** some papers: from 20 % to less than 40 %, **** many papers: more than 40 %)

The scale and number of local case studies are disproportionate when compared with the number of metadata analyses and reviews. The number of papers taking a multilevel approach is particularly low. Climate change policy is usually determined at the national level, while adaptation mostly takes place at the local level. Adaptation and vulnerabilities are known to be mostly local in nature, and the complexity of gender relations at the local level calls for more context-specific data, in order to draw solid conclusions for research, development and policy-making. Further efforts must be made to understand these parallel realities, as any climate change policy, plan or programme affecting natural resource management, agriculture production or the energy sector, will affect gendered access patterns, division of labour, health and income, and will therefore impact both vulnerabilities and gender relations.

### Why gender? Gender rationale in the vulnerability analysis

Differences in perception were found to be one of the rationales for the gender analysis. Men and women are reported in only a few papers to have different perceptions of climate variations, their causes and impacts. The studies build their gender rationale on the assumption that differences in perceptions will result in different responses to climate change (Dankelman 2002; Nelson et al. 2002; Shaffer and Naiene 2011; Safi et al. 2012). Many papers focus only on the perceptions of climate variations, rather than their implications for the vulnerability of individuals or households (Ofuoku 2011; Shaffer and Naiene 2011; Cherotich et al. 2012; Oyekale and Oladele 2012; Safi et al. 2012; Sanchez et al. 2012; Boissière et al. 2013). Some studies also explored differences in perceived needs related to adaptation (Cherotich et al. 2012; Oyekale and Oladele 2012). No significant differences in perception were found by Sanchez et al. (2012) or Boissière et al. (2013). In many papers on perception, such differences are explained by gendered livelihood activities, roles and responsibilities, and are sometimes also justified by general statements about women’s vulnerability.

Finding how gendered differences affect responses to climate change impacts is the rationale behind the integration of gender in some studies. The most common rationales include the following: higher rates of death among poor women and children due to air pollution caused by household fuel (Venkataraman et al. 2010); skipping meals and reducing food intake on a regular basis (Beaumier and Ford 2010); and socio-economic stresses and gendered health impacts (Dean and Stain 2010). The role of women in producing and providing food for their households in areas at high risk of climate variations and conflict was also mentioned as an underlying principle for the relevance of gender (Cherotich et al. 2012). The lack of women’s participation in development is an additional rationale for integrating gender analysis in the studies. Some studies justify the consideration of gender in climate change by citing the limitations on women’s participation in the implementation of adaptive strategies, e.g. due to their exclusion from access to land and water (Nation 2010). Other studies justify the inclusion of gender in more general ways, such as by emphasizing the need to gain a deeper understanding of issues that are critical to community members (Cassidy and Barres 2012), or the failure of previous interventions because of a lack of gender integration in their implementation (Nielsen et al. 2012). Few papers refer to women’s agency, active choices and engagement (Gabrielsson and Ramasar 2013; Jerneck and Olsson 2013). Inequality in general, and the gender aspects of inequality, are seldom addressed sufficiently in the case studies. Only a very few papers use equity and rights-based arguments when examining gender integration (e.g. Onta and Resurrección 2011).

### Gender-disaggregated analysis, intersectionality and the ‘Olympics of vulnerability’

To understand how social determinants intersect and how power relations impact on inequities and create vulnerability, our analysis shows that very few papers addressed or conducted an explicitly designed analysis of power relations, including context-specific mechanisms of exclusion and marginalization. Eriksen et al. (2005), however, do address power relations, and use a feminist political ecology framework to analyse vulnerabilities. Some papers acknowledge the importance of gender as a significant source of power differentials, but choose not to focus on the analysis of power relations. Very few papers took a self-examining approach, and reflected on their own role, and the unbalanced relationship between researchers and communities (Beaumier and Ford 2010).

Our results indicate that none of the articles’ authors identified their focus as being intersectional, and that the intersectionality framework is not yet embedded, and perhaps is even entirely absent from the scientific literature on climate change. Despite the lack of an intersectional framework, a few papers refer clearly to existing intersectional inequalities (Onta and Resurrección 2011; Andersson and Gabrielsson 2012; Djoudi et al. 2013) and acknowledge that gender interacts with ethnicity, caste and class. Of the papers that do not refer explicitly to the intersectionality as a conceptual framework, only a few take into consideration the existence of subgroups within male and female groups. Lama and Dalit women in Nepal are primarily responsible for agriculture. However, Dalits must depend on the leasing of Lama land in order to cultivate their crops and must give half the yield to the landowner and seeking day-labour jobs in return for in-kind compensation (Onta and Resurrección 2011). A study conducted in Burkina Faso demonstrates that Fulbe women’s adaptive capacities to climatic irregularities were impacted by the fact that they often live in isolation from other households. By contrast, Rimaiibe women work collectively to lessen responsibilities, thus giving themselves time to develop a skill and bring a steady income to the household (Nielsen and Reenberg 2010). In Mali, study participants, depending on their gender, age, class, ethnicity and practice, described varied adaptive strategies. For example, pastoral communities saw seasonal herding of livestock as the most important strategy, whereas agricultural farming communities did not share the same priority. Adaptive strategies differed, as women from the pastoral community were keepers of sheep and goats, whereas women from the agricultural farming community invested in charcoal production (Brockhaus et al. 2013).

Although they do not formally use intersectionality as a framework, many papers disaggregate data according to different social categories in their analysis. We analysed the papers to identify the social categories included in the vulnerability analyses. The results indicated that most papers use two categories (i.e. men and women), while the other papers include at least one other social distinction in their analysis. Most of the papers that consider factors other than just the fact of being either women or men in the analysis include age, a few include ethnicity, and a very few include profession and wealth. However, it is important to highlight that the majority of the papers that refer to those categories do so almost exclusively in their statistical data analyses but just as characteristics of household variables.

For the few papers that address intersectional gendered dynamics in their results section, their conclusions were less likely to capture these findings, as recommendations were aimed at the community level. Onta and Resurrección (2011) and Djoudi and Brockhaus (2011) draw attention to gender and caste intersections, providing new evidence of possibly unintended gender outcomes that are pushing and challenging ethnic, caste and gender social barriers.

However, despite the relatively large number of papers that include a range of social categories, at least in their analysis of data, the intersectional nature of vulnerability is rarely addressed. In addition to this, most papers do not use a gender framework based on contextual, power relations analysis, and disaggregate data later according to gender, age, wealth, etc.

Without embedding itself in societal, local and global inequalities and power relations analysis, research runs the risk of being reduced to a metaphor by simply pointing out the most vulnerable. We argue that current debates and analyses on climate change vulnerability are in line with Martinez’s (1993) concept of ‘oppression olympics’, and could perhaps be referred to as ‘vulnerability olympics’. This outcome is unsurprising, as it is far simpler for agencies and donors to target the most vulnerable than to understand and generate transformational change.

### From female-headed households to women’s vulnerabilities

A large number of vulnerability papers agree that assets and context promote vulnerability and barriers to adaptation for women. We found that most articles that analyse vulnerabilities at the local level use a female-headed household approach. The sampling is mostly random, and the analyses use the proportion of female-headed households to male-headed households. The greater vulnerability of female-headed households is shown in few studies (Cassidy and Barres 2012; Maponya and Mpandeli 2012; Safi et al. 2012). Different factors are identified to explain the greater vulnerability of female-headed households. Generally, female heads of household are found to have a lower level of education than male heads of household. The gender and level of education of the head of household are closely correlated, and are cited as a possible explanation for differences in vulnerability (Deressa et al. 2009 Below et al. 2012). Other studies argue that a lack of formal education and the social standing of female heads of household were found to limit access to credit (Below et al. 2012; Banerjee et al. 2013) and to increase therefore the female-headed households’ vulnerability. In some studies, female-headed households experience greater vulnerability because, in contrast to most male-headed households, they usually lack reliable, non-farm income (Eriksen et al. 2005; Antwi-Agyei et al. 2012). Female-headed households are also often on the edges of their community’s social network, which limits their engagement in resilient livelihood strategies and which was found to impact on the resilience of the entire household (Cassidy and Barres 2012). However, the existing evidence on the comparative vulnerability of female- and male-headed households is not sufficient to draw strong conclusions that one is worse-off or better-off than the other. In particular, the heterogeneity of the vulnerability context considered in the studies is a limiting factor for unambiguous and strong conclusions.

The results on the ‘feminization of vulnerability’ of households show similar patterns to previous controversial statements on the feminization of poverty and its link to the feminization of a household’s headship (Chant 2003; Aurora-Jonson 2011). We recognize through our findings a continuum of those generalized assertions in the gender and climate change literature. As noted by Chant (2003), statements linking the feminization of poverty with the feminization of household headship draw their legitimacy from the cumulative repeating and grafting of those discourses into the literature on development. We argue that this perseverance of the association of poverty with the feminization of a household’s headship has also certainly legitimized the discourse of vulnerability and the feminization of heads of households.

Very few papers differentiated intra-household vulnerabilities (Nation 2010; Andersson and Gabrielsson 2012). Nation (2010) was one of the few authors to highlight the crucial importance of examining intra-household dynamics and livelihood strategies, as well as the political, economic, social and natural environments in which the household is embedded, to understand gender vulnerabilities.

In the remaining papers, conclusions were not straightforward: women and men were found to have different assets and face different contextual constraints. None of the papers attempted to provide a summary of their findings as a simple comparison of the vulnerability of men and women, due to the multiple dimensions of vulnerability and the diversity of situations (Eriksen et al. 2005; Antwi-Agyei et al. 2012; Kisauzi et al. 2012; Mogotsi et al. 2012).

In short, most studies that conclude that women are more vulnerable are based on comparisons between female- and male-headed households. An interesting shift, however, occurs in the vulnerability discourse, moving from the evidenced female-headed household vulnerability towards a generalized women’s vulnerability.

In terms of impact, we found that few papers indicate that women and men are impacted differently by climate variability. Furthermore, of the articles that indicated that women are more vulnerable than men (Wilk and Kgathi 2007; Nation 2010; Bokhoree et al. 2012; Cassidy and Barnes 2012; Cherotich et al. 2012; Drolet 2012; Maponya and Mpandeli 2012; Safi et al. 2012), only a very few refer to their own data, that is, to their own evidence. The remaining statements were based on theoretical backgrounds and analysis. Although the vulnerability of women appears to be obvious—because of the social, political and economic marginalization experienced by women in various contexts—little is known about the local gender dynamics of vulnerability. There is a lack of data and local context analysis to reinforce and elucidate these assumptions.

### Adaptation as emancipation: How resilient are social and gender boundaries?

In order to understand the transformational processes of adaptation, we aimed to analyse (using the intersectionality framework) whether, and to what extent, adaptation processes reinforce or challenge hierarchical, unequal social structures. We analysed articles and case studies that document changes in social structures associated with environmental change and adaptation to it. Six papers documented a social shift in relation to gender as a consequence of adaptation to climate change (Andersson and Gabrielsson 2012; Nielsen and Reenberg 2010; Onta and Resurrección 2011; Ford and Goldhar 2012; Nielsen et al. 2012; Djoudi and Brockhaus 2011). In a study conducted in Burkina Faso, female participants stated that the empowerment discourse of some adaptation projects enhanced their capacity to negotiate and obtain new roles within the household (Nielsen and Reenberg 2010). Djoudi and Brockhaus (2011) find that women from socially disadvantaged groups were able to engage in new activities after the social structure of their community was affected by the impacts of drought and most adult men had migrated. They were less vulnerable than women from the higher social class, who were restricted in their mobility and in the strategies they could adopt to cope with environmental change. Ford and Goldhar (2012) describe similar patterns of women moving into salaried positions. Those women emphasized increasing freedom of choice in recent years, which they associated with environmental changes. The same pattern was identified by Nielsen et al. (2012), who emphasize the importance of changes in livelihood activities, and the connection between wage labour and greater economic freedom for women. Those authors argue that such shifts have contributed to a change in gender roles for women and young people, which was apparent in the greater role played by women in household decision making. Andersson and Gabrielsson (2012) found that environmental pressures created shifts in social patterns and how the agency of widows improved through those shifts. According to the authors, this led to many achievements, including the prevention of crop failure, reduced workload, increased nutritional intake, increased sustainable water management, diversified and increased income, and improved strategic planning. Onta and Resurrección (2011) find that environmental changes and the processes of adaptation in India resulted in new social interactions, including shifts in traditional caste structures. New social dynamics, including shifts in gender patterns in response to multiple stressors, have also been identified in Kenya and Uganda (Folmar 2007).

However, other studies do not confirm this emancipatory trend, and show that in times of crisis, social structures and power relations can be very resistant to change. Carr (2008) argues that, in spite of their unequal and less-than-optimal material outcomes, maladaptive pathways persist because they are rooted in the ability of men to legitimize and reinforce a link between adaptation and existing unbalanced gender roles. Andersson and Gabrielsson (2012) emphasize that adaptation processes based on collective action can produce positive gender outcomes. Although gendered norms largely remain, women’s engagement in collective action has contributed to strengthening their bargaining and decision-making power within the household. However, although collective action can empower women and marginalized groups, existing power structures can limit such groups’ access to various resources, and thereby reduce their impact (Ballet et al. 2007; Pandolfelli et al. 2008).

### Women’s strategies: The feminization of male-dominated sectors?

Many papers indicate that men and women have different coping or adaptation strategies. Few papers focus on one specific adaptation activity, and suggest that men and women play different roles in the implementation of a given adaptation (Eriksen et al. 2005; Ziervogel et al. 2006; Cassidy and Barnes 2012; Molua 2012; Pangapanga et al. 2012). In Mozambique and Mali, migration was identified as a clearly male-dominated strategy (Silva et al. 2010; Djoudi and Brockhaus 2011; Brockhaus et al. 2013; Djoudi et al. 2013). In South Africa, differences were reported in the strategies adopted by men and women. For instance, women were more informed than men in their agricultural choices, on issues such as suitable crops for home consumption and trade (Ziervogel et al. 2006). In Tanzania and Ethiopia, women were found to be less engaged in adaptation practices based on tree plantations (Deressa et al. 2009; Below et al. 2012). Among teenagers psychologically affected by droughts in Australia, girls have significantly higher levels of prosocial behaviour than boys (Dean and Stain 2010). In Malawi, there are gender differences related to the choice of improved varieties, shifting of planting dates, irrigation farming, and income-generating activities (Pangapanga et al. 2012). In Cameroon, female-headed households are less likely to plant trees (as a protective measure) or rebuild homes (Molua 2012). In Kenya, gum and resin collection is one of the most popular strategies adopted by women, children and impoverished people (Gachathi and Eriksen 2011).

The diversification of livelihood activities is known to be an important strategy for reducing household vulnerability, particularly for activities that are not weather dependent. Several studies document the gender limitations to diversifying activities and livelihoods. However, the link between the available choices for different groups and gendered social roles and norms has not been well assessed. Few studies refer to context-specific, cultural limitations or to the options that women and female-headed households are culturally allowed to choose and implement. The prevalence of socially driven gender inequity is reflected in the relatively limited choices and strategies that many women feel that they are allowed to adopt. Some studies document that female-headed households are often constrained to engaging in low-benefit, low-risk activities, due to their exclusion from high-benefit, low-risk activities, such as formal employment, which are mostly male dominated (West et al. 2008; Molua 2012). Djoudi and Brockhaus (2011) suggest that long-term strategies for women are based on education and formal employment, but that short-term community strategies hinder them from making the shift away from high-risk, low-benefit strategies. Education seems to ensure access to better incomes and provide access to other assets required to adapt, as many studies suggest. The probability of selecting resilient pathways is highest for an educated, middle-aged, male farmer, while female-headed households face significant socio-economic and cultural constraints, which limit their ability to choose resilient strategies (West et al. 2008; Molua 2012). Pastoralism and migration are both important strategies in several drought-prone regions, and are more resilient strategies than rainfall-dependent agriculture. Both sectors are mostly male dominated (West et al. 2008; Brockhaus et al. 2013).

Adaptive strategies with gender-differentiated outcomes were cited in some papers, providing further analysis of the gender-differentiated impacts of adaptation strategies. The negative impacts of some strategies on women were described in some studies. Nation (2010) documents that in Senegal, the introduction of irrigation technologies to adapt to drought increased many women’s dependency on men and male control, in contrast to the traditional cultivation system, which allowed women to be independent, own-account farmers. Under this traditional system, their work was neither supervised nor dictated by males. In Mali, Djoudi and Brockhaus (2011) found that women belonging to a higher social class were culturally not allowed to take over male-dominated activities after men had migrated.

According to several papers, migration is a male-dominated strategy expected to impact gender relationships. The question of whether the migration of men has a negative or positive impact on gender relationships is controversial and certainly context dependent (Hecht et al. 2015). Some studies have documented the impacts of migration on the feminization of traditionally male-dominated sectors, for instance, livestock and pastoralism. The livestock mix follows a trend towards a greater number of small ruminants and fewer cattle (Sungno Niggol and Mendelsohn 2006). This trend is associated with drought-related adaptation and results in more women being involved in the livestock sector (Turner 1999). This is a good example of how the strategies of men, in a context of unequal decision making, can have gender-mixed and complex outcomes. Hence, further studies are required to examine the feminization of male-dominated sectors, as a result of coping and adaptation strategies.

## Discussion

Our first finding shows that gender is addressed less frequently in studies on mitigation than in those on adaptation. Mitigation studies focus on technical solutions and measures. Although none of the studies explicitly make the connection of women as ‘adaptors’ and men as ‘mitigators’, we argue that—especially in light of the disproportionate number of articles dealing with gender in adaptation and mitigation—such a trend would be expected as a continuum of the dichotomies that characterize and nurture social perceptions of masculinity and femininity. MacGregor (2010) highlights that by ‘scientizing and securizing’ (MacGregor 2010, p. 128) the debate on climate change, the solutions expected are constructed around the traditional male-dominated domain. The consequence is that the climate change domain is dominated by men in the research, policy, implementation and advocacy arenas, as highlighted in several studies (Rosa and Dietz 1998; Dankelman 2002; MacGregor 2010). In order to avoid the exacerbation of existing patterns, and the creation of new vulnerability patterns, we agree with MacGregor’s (2010) call for more studies on the sociology of climate change and for a stronger consideration of feminist research on climate change.

In the absence of a specific gender and climate change framework, it is very difficult for most studies to draw comparative conclusions on either the gendered aspects of vulnerability, or on the differentiated impacts and gendered outcomes of climate and environmental change.

The concept of gender was applied in a very uneven manner. A large number of the papers included more categories than just gender in their analysis, although none of the papers performed an identifiable, clear, intersectional analysis. Hence, in terms of the dimensions of intersectionality that we considered (i.e. intersectional categories, multilevel analysis, power, and emancipation), few conclusions taking intersectionality considerations into account were provided in these papers. Age and ethnicity were also included as categories in most papers, but more as an explanatory variable in statistical models than as a determinant of power and inequity. Most papers used metadata, and the case studies mainly focused on one level of analysis. Very few papers analysed the power relations that produce inequities. Some studies demonstrated that in some contexts, through the process of adaptation, men and women are challenging gender and caste rules. This could be an indication of emancipatory trajectories, which require further study in the context of adaptation.

Evidence on gender vulnerability at the local level is limited, and much of what is known is based on surveys of female- and male-headed households. Generally, the studies performed random sampling and then used social determinants, such as age, wealth, and gender of the households in their analysis. Most of the studies report having a greater number of male respondents than female. Many of these studies indicate that female-headed households are more vulnerable than male-headed households. However, in other papers, conclusions are not so straightforward. Some studies suggest that although women and men have different assets, and face different contextual constraints, it is unhelpful to summarize these differences as a simple comparison of the vulnerability of men and women, due to the multiple dimensions of vulnerability and the diversity of contexts. We argue that it is difficult to make broad comparisons, particularly in the absence of a gender framework or contextual power analysis (Eriksen et al. 2005; Antwi-Agyei et al. 2012; Kisauzi et al. 2012; Mogotsi et al. 2012). Most householder characteristics relevant to vulnerability are not independent, but are related, such as education and gender.

Many questions arise regarding the nature of the confirmed vulnerability of female-headed households. Are certain households more vulnerable because they are headed by a female member of the family, or is their vulnerability caused by the same factors that caused them to be headed by a woman? In most cultural contexts, female heads of household are the result of a social process or change, such as the death, migration, or illness of the male head of household. In a few cases, women became household leaders through an emancipatory process (Deressa et al. 2009). We argue that vulnerability analysis should go beyond an approach that compares female- versus male-headed households, to addressing the structural causes of vulnerability. For instance, studies on cultivating tree plantations as an adaptive strategy suggest that female heads of household adapt less readily. In many cultural contexts, tree plantations are a male domain; here, the root cause of the female heads of households’ inability to adapt may have been due to their restricted use of this tree plantation strategy. These women may, however, have developed emancipatory spaces to adopt other solutions. It is important to better understand such differentiated vulnerabilities, in light of specific patterns of power and agency, as well the negotiations and dynamic nature of gender relations.

An interesting shift occurs, however, in the vulnerability discourse when it conflates the vulnerability of female-headed household with a more generalized and unsubstantiated claim of women’s vulnerability. Hence, the discourse then moves from female-headed household vulnerability towards a generalized women’s vulnerability. This shift moves towards the assumption that women are a homogeneous group as indicated by Arora-Jonsson (2011), despite earlier feminists’ work rejecting the essentialist and universal notion of women as a homogenous group (Jackson and Pearson 2005). This however is one of the most misguided assumptions in climate change and gender research, as it ignores the crucial importance of other social factors and does not acknowledge the specific social context of female-headed households. However, this assumption might be induced by the general misleading perception of the concept of vulnerability, as associated with passive, innocent, victimhood (Alaimo 2012). Thus, this author calls for the term “insurgent vulnerability” to be used, defining it as an understanding of vulnerability “that does not entrench gender polarities but instead endorses biodiversity, cultural diversity, and sexual diversity” (Alaimo 2012, p. 10).

Although most studies carried out interviews (mostly with female- vs. male-headed households) and participatory workshops, their conclusions on gender are mainly based on household surveys. In some cases, gender-relevant qualitative information gathered through participatory workshops was not included in the papers’ conclusions. Most recommendations were aimed at the community or household level, and called for: greater assistance for female-headed households; targeted climate adaptation policies and programmes to enhance asset building and increase the capacity of vulnerable households to engage in more resilient non-farm activities (Antwi-Agyei et al. 2012); and greater financial inclusion and access to formal systems of finance (Banerjee et al. 2013). Many studies also recommended the improvement of education systems to provide equal opportunities for women, and the strengthening of social capital, agricultural extension, microcredit services, and access to information (Below et al. 2012; Kisauzi et al. 2012). Very few studies called for approaches and strategies that recognize people’s agency (Brown 2011). In addition to this, very few papers called for greater understanding of intra-household vulnerability and vulnerability dynamics (Nation 2010), or targeted a specific group of vulnerable women at the household level in their recommendations (Gabrielsson and Ramasar 2013). Those papers, that did, concluded that policymakers and practitioners must consider the intra-household distribution of labour and resources, as well as agricultural and other household activities, in order to understand women’s vulnerabilities and adaptation processes. Wilk and Kgathi (2007) conclude their study by highlighting that there are both social and spatial differences in risk and these need to be better understood by policy makers in order to better target initiatives. Jerneck and Olsson (2013) call for inclusive and participatory processes, which reconsider and act upon the underlying, structural layers of poverty and the need for broader policies on social change.

Many of the studies included gender as a variable in their analysis, but no gender-specific recommendations are made in their conclusions (Deressa et al. 2009). Carr (2008, p. 298) questions “what is successful adaptation, since socially just outcomes would result in unacceptable challenges to men’s authority, and how might we foster adaptation that leads to both social justice and material security”. Some considered gender in their methods (e.g. by working with male and female focus groups), but provided few gender comparisons. These studies gathered the views and opinions of different stakeholders, but did not make comparisons between the coping and adapting strategies of men and women, nor between the different barriers to adaptation that they face (Ziervogel et al. 2006; West et al. 2008).

## Conclusion

Similar to early research on forests and gender, and food security and gender, the work on climate change and gender continues to ignore structural inequalities and gendered power relations. The broader literature on climate change adaptation and vulnerability pays little if any analytical attention to power relations, and almost none to gendered ones.

While there is a call for further study on the differential impacts of climate change, there is little evidence or research to support claims about gendered vulnerabilities, or about the various coping and adapting strategies of different social groups. Most of the articles that addressed different social groups took an additive approach, rather than perform a deeper analysis of vulnerability by investigating how different social statuses intersect.

Some studies showed great awareness of the importance of understanding multiple statuses. The implementers of such studies should be encouraged to take their analysis further by considering how these statuses intersect to create a vulnerability context. In the words of Arora-Jonsson (2011, p. 750) “A feminist response to global climate change must not only challenge masculine technical and expert knowledge about climate change, it must also question the tendency to reinforce gendered polarities, which work to maintain the status quo”.

One objective of this paper was to set up an intersectional perspective for gender analysis in climate change. However, because very little of the reviewed literature offered an intersectional analysis, we encountered some limitations when applying the intersectionality framework for broader social categories. However, we highlighted some specific results, including more social categories for men and women (ethnicity, age, race, etc.), whenever relevant data and studies were available.

The results on perception in this study were mixed, and it is difficult to draw solid conclusions on how the gendered perceptions of risk influence adaptation. Further analysis is required to understand differences in perception in a broader gender context. We argue that rather than being an inherent difference, perceptions reflect inequalities in many ways. In fact, the causes and effects of vulnerability are twofold and commutable. Furthermore, economic and physical marginalization, and marginalization more generally, are highly relevant to perceptions of risk. Men who are exposed to discrimination and feel vulnerable have higher perceptions of risk (Finucane et al. 2000). For example, research has shown that white males have a relatively low perception of climate risk, which is known as the ‘white male effect’ (Finucane et al. 2000). However, recent studies in countries where inequalities between men and women are less prevalent have challenged this view by identifying no significant differences in perception between men and women (Olofsson and Rashid 2011). Several scholars have called for further study on differences in risk perception between various groups, to be carried out in a less deterministic way using an inequality lens. We agree that the societal inequality effect is a more likely explanation for divergent perceptions, than inherent differences between men and women. Several papers included in this analysis went further than a deterministic interpretation of men’s and women’s divergent perceptions of climate variability. They attribute those differences not to inherent, fundamental, and natural differences between females and males, but rather to the context of inequity in which those perceptions were established and shaped. For example, Cherotich et al. (2012) and Safi et al. (2012) argue that women may perceive the risk of environmental change to be more acute due to a lack of gender equity and differentiated political power. They reinforce their findings by citing studies in countries with greater gender equity, where no difference in the perception of risk was found (Olofsson and Rashid 2011).

This paper argues that understanding the gendered effects of climate change requires critical assessment. The current understanding and analysis of gendered vulnerability is far removed from what Alaimo (2012, p. 10) calls “insurgent vulnerability”, defined as a type of vulnerability “that does not entrench gender polarities but instead endorses biodiversity, cultural diversity, and sexual diversity, and recognizes that we all inhabit trans-corporeal interchanges, processes, and flows”. This will require a paradigm shift from viewing gender as just an empirical category (men vs. women), towards carrying out an intersectional gender analysis. This would include an understanding of the discursive construction of gender and the analysis of power relations that shape the perception of the vulnerability and responses to the environmental, the sociological, economic and political impacts of climate change.

In this sense, we argue that gaps identified by this study related to the lack of intersectional approaches as well as to a lack of power relation analysis in the climate change debate can be addressed by a stronger inclusion of feminist theory into the field of climate change (Sultana 2014). In the words of MacGregor (2010, p. 137), “It is also important that materialist-informed empirical research be complemented by critical feminist theorizing of non-material and discursive aspects of climate change”. The concept of gender and climate change needs to “challenge embedded assumptions about gender and power”, and to make “new alliances out of old divisions” (Cornwall 2003, p. 1325). Furthermore, it needs to move beyond adjustment measures by contextualized understanding of mutual fragility (Tschakert and Machado 2012) towards an inclusive and transformational culture and practices. If we ignore the social and political foundations that have contributed to climate change vulnerability and the ensuing climate change crisis (Wainwright 2010), potential solutions will enhance rather than reduce existing injustices, and societies risk missing the opportunity to address the critical challenges of climate change.
